# Supplementary figures and images for: The Order of Trait Emergence in the Evolution of Cyanobacterial Multicellularity
Source: Genome Biol Evol. 2020 Nov 24;13(2):evaa249. doi: 10.1093/gbe/evaa249 (PMC7937182; doi:10.1093/gbe/evaa249)

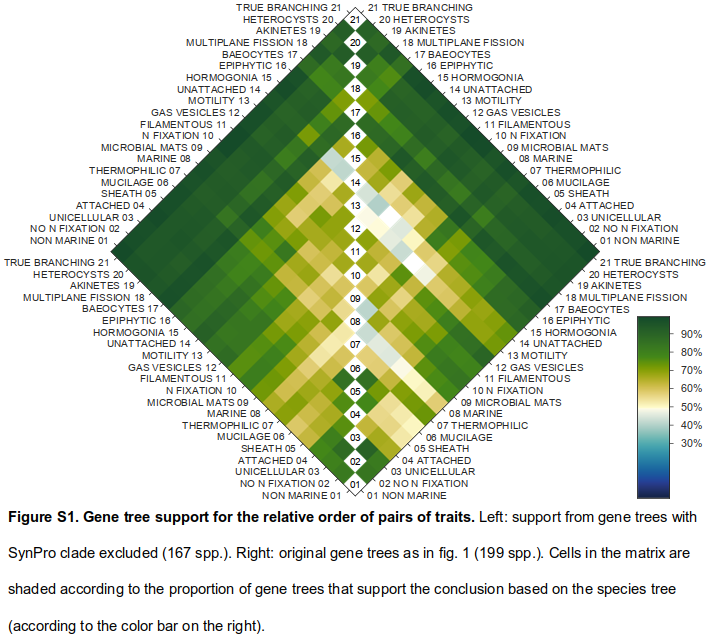

Supplement: evaa249_Supplementary_Data [file evaa249_supplementary_data.zip › fig s1.png]
